# Machine learning improves detection of alpha thalassemia carriers compared to clinical features

**DOI:** 10.1038/s41598-025-20605-6

**Published:** 2025-10-21

**Authors:** Elmira Mohammadi, Mohsen Rastegar, Amir Jamshidnezhad, Amirabbas Azizi

**Affiliations:** 1 Thalassemia & Hemoglobinopathy Research Center, Health Research Institute, Ahvaz Jundishapur University of Medical Sciences, Ahvaz, Iran; 2 Student Research Committee, Ahvaz Jundishapur University of Medical Sciences, Ahvaz, Iran; 3 Department of Non-Communicable Diseases (Genetics), Shiraz University of Medical Sciences, Shiraz, Iran; 4 School of Allied Medical Sciences, Ahvaz Jundishapur University of Medical Sciences, Ahvaz, Iran

**Keywords:** Alpha-thalassemia, Machine learning, CRISP-DM, Predictive modeling, Thalassemia screening, Genetic testing, Data mining

## Abstract

Alpha-thalassemia is a widespread genetic disorder, and accurately distinguishing between alpha-plus (α⁺) and alpha-zero (α⁰) types is critical for effective screening and management. This study developed and evaluated machine learning models to classify α⁺ and α⁰ carriers based on hematological parameters. A dataset of 956 cases was analyzed, including variables such as red blood cell (RBC) count, hemoglobin (Hb) level, and RBC indices. Feature selection identified the most predictive markers, and five machine learning models were trained and compared. The stacking ensemble model demonstrated the best performance, achieving 94% accuracy and a high F1-score. Key predictors included RBC count, mean corpuscular volume (MCV), mean corpuscular hemoglobin (MCH), and mean corpuscular hemoglobin concentration (MCHC). Correlation analysis revealed strong interrelationships among RBC indices, while platelet (PLT) and white blood cell (WBC) parameters had moderate associations. These findings suggest that machine learning, particularly ensemble methods, can enhance the detection of alpha-thalassemia carriers. The development of models based on both data-driven and clinical features provides a flexible framework for screening and could support more personalized approaches in future research.

## Introduction

Thalassemia is a significant global health challenge, with around 5% of the world’s population carrying at least one α-thalassemia mutation. In certain regions of Southeast Asia, carrier rates are even higher, reaching 80% to 90%^1^. In Iran, approximately 2 million people are carriers, with the highest prevalence found in the northern and southern regions, where α-thalassemia affects about 35% of the population^2^. A major contributing factor is the high frequency of consanguineous marriages, common among various ethnic groups^3^. Additionally socio-cultural, healthcare-related, and economic barriers such as limited access to genetic counseling, insufficient prenatal diagnostics^4–6^, and inequities in healthcare services^7^ further contribute to the persistence of thalassemia. Moreover, the disease imposes a substantial economic burden, not only through direct medical costs but also through indirect effects such as diminished quality of life, reduced productivity, and psychosocial challenges for both patients and society. Due to its chronic nature and the lifelong need for medical care, thalassemia requires significant healthcare resources for effective management and treatment^8–10^. Addressing these issues requires comprehensive genetic screening programs and effective public health interventions.

Machine learning is increasingly used in hematology to improve diagnostic accuracy, automate complex and time-consuming tasks, and help reduce healthcare costs related to diagnostics, treatment, and drug development^11–13^. machine learning techniques now support various stages of patient care, from predicting diagnoses using blood test results to guiding personalized treatments based on genetic data^14^. Both machine learning and deep learning (DL) have been applied in the classification, screening, diagnosis, and management of various blood disorders, including anemia^15^ and hematologic cancers^16^. These methods leverage multiple data sources, such as genomic information, histopathology images, sensor-based data, routine blood test results, flow cytometry data, or combinations of these inputs^17^.

Although prior studies have explored the application of artificial intelligence (AI) and machine learning in thalassemia diagnosis, they have primarily focused on distinguishing beta-thalassemia from iron deficiency anemia and identifying individuals with thalassemia or other hemoglobinopathies from healthy individuals^18–22^. The focus on beta-thalassemia may be due to its greater clinical severity and significant public health impact. However, less attention has been given to classifying alpha-thalassemia subtypes. Distinguishing alpha-plus (α⁺) from alpha-zero (α⁰) mutations is challenging because their hematological profiles overlap, and mutation patterns vary by population. This highlights the need for models that can accurately classify alpha-thalassemia mutations using local genetic and hematological data to better identify carriers.

This study aims to address this gap by developing a highly accurate machine learning model for detecting deletion mutations in alpha-globin genes among alpha-thalassemia patients. Using locally sourced clinical data, the research focuses on identifying key blood parameters, selecting the most effective machine learning model, and evaluating its performance across both feature-driven and clinically based models. By leveraging advanced machine learning techniques and a structured data mining approach, this study introduces a practical framework to enhance alpha-thalassemia diagnosis and ultimately support clinical decision-making in regions with high disease prevalence.

This paper is organized as follows: Section ‘Methodology’ details the CRISP-DM methodology and implementation stages. Section ‘Results’ presents the study’s findings, followed by Section ‘Discussion,’ which interprets these findings and compares them with prior studies while addressing how limitations affect their generalizability. Finally, Section ‘Conclusions and Future Work’ summarizes key insights and suggests directions for further research.

## Methodology

**Fig. 1:**
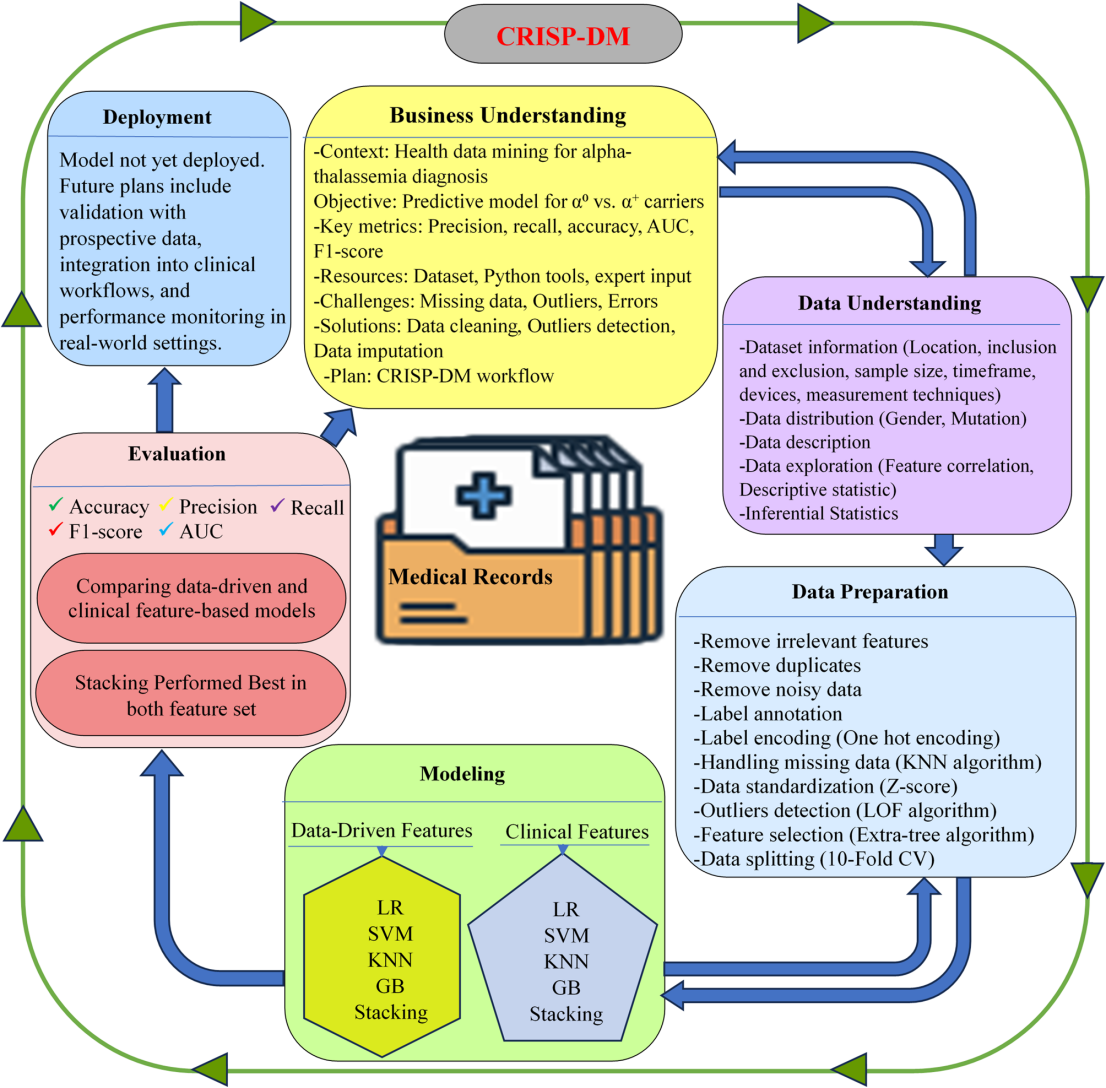
Workflow Framework for the Detection of Common Large Alpha-Thalassemia Mutations Using Machine Learning.

This study followed the Cross Industry Standard Process for Data Mining (CRISP-DM) as the main framework for project development. CRISP-DM is a well-structured methodology that has been widely used since the 1990s in both Knowledge Discovery in Databases (KDD) and modern data science projects^23^. Its popularity comes from its simplicity, repeatability, and effectiveness in guiding data mining projects^24^. CRISP-DM is also valued for being intuitive, adaptable, and closely aligned with standard data science workflows^23^. By ensuring a clear connection between project goals and technical implementation while allowing iterative improvements, it was an ideal choice for this study. The project followed the six CRISP-DM phases: business understanding, data understanding, data preparation, modeling, evaluation, and deployment^25^ as shown in Fig. 1.

###  Business understanding

This project was developed within the context of health data mining to improve diagnostic support for alpha-thalassemia carriers by analyzing patient laboratory data. The main goal was to develop a predictive model that would classify patients as carriers of either α⁰ or α⁺ thalassemia using routine hematological indices. The technical objective was to create a model with high predictive performance, evaluated by metrics such as precision, recall, and overall accuracy. During the initial phase, available resources including the dataset, computational tools (Python and associated libraries), alongside consultation with a genetics expert, were assessed. Key challenges, such as missing data, outliers, and noise were identified, with planned solutions like data cleaning, outlier detection and data imputation techniques. A structured CRISP-DM workflow diagram (Fig. 1) was created to outline the methodology and serve as a step-by-step execution plan.

###  Data understanding

This phase involves data collection, examination, and exploration to detect quality concerns, meaningful patterns.

#### Data collection

- Informed consent.

This retrospective study was approved by the Ethics Committee of Ahvaz Jundishapur University of Medical Sciences (AJUMS) under approval number IR.AJUMS.REC.1402.311, and the requirement for informed consent was waived in accordance with national regulations due to the anonymized nature of the data. All records were de-identified, and strict confidentiality and data protection standards were maintained throughout the study.

- Compliance with guidelines.

All methods were performed in accordance with the relevant guidelines and regulations stipulated by the Ethics Committee of Ahvaz Jundishapur University of Medical Science (AJUMS) and local data protection laws.

- Research environment.

The data were collected from the Thalassemia and Hemophilia Research Center and Genetics Laboratory located at Dastgheib Educational and Medical Center in Shiraz. The center’s services include chorionic villus sampling (CVS), thalassemia prevention testing, fetal screening, coagulation factors and inhibitors testing, male infertility testing, and amniocentesis.

- Inclusion and exclusion criteria.

Inclusion criteria required patients to have documented common large deletions in the alpha-globin gene, including heterozygous α ^3.7^, heterozygous α^4.2^, homozygous α^4.2^, large deletions such as − α^20.5^ and −−^Med^, or compound heterozygous mutations (α ^3.7^/ α^4.2^). Additionally, patients had to have HbA2 levels below 3.5%. Exclusion criteria included cases where marriage counseling was discontinued, records that were corrupted or illegible, incomplete data due to sample depletion, follow-up loss resulting in missing or incomplete records and cases with coexisting iron deficiency.

- Data distribution.

**Fig. 2:**
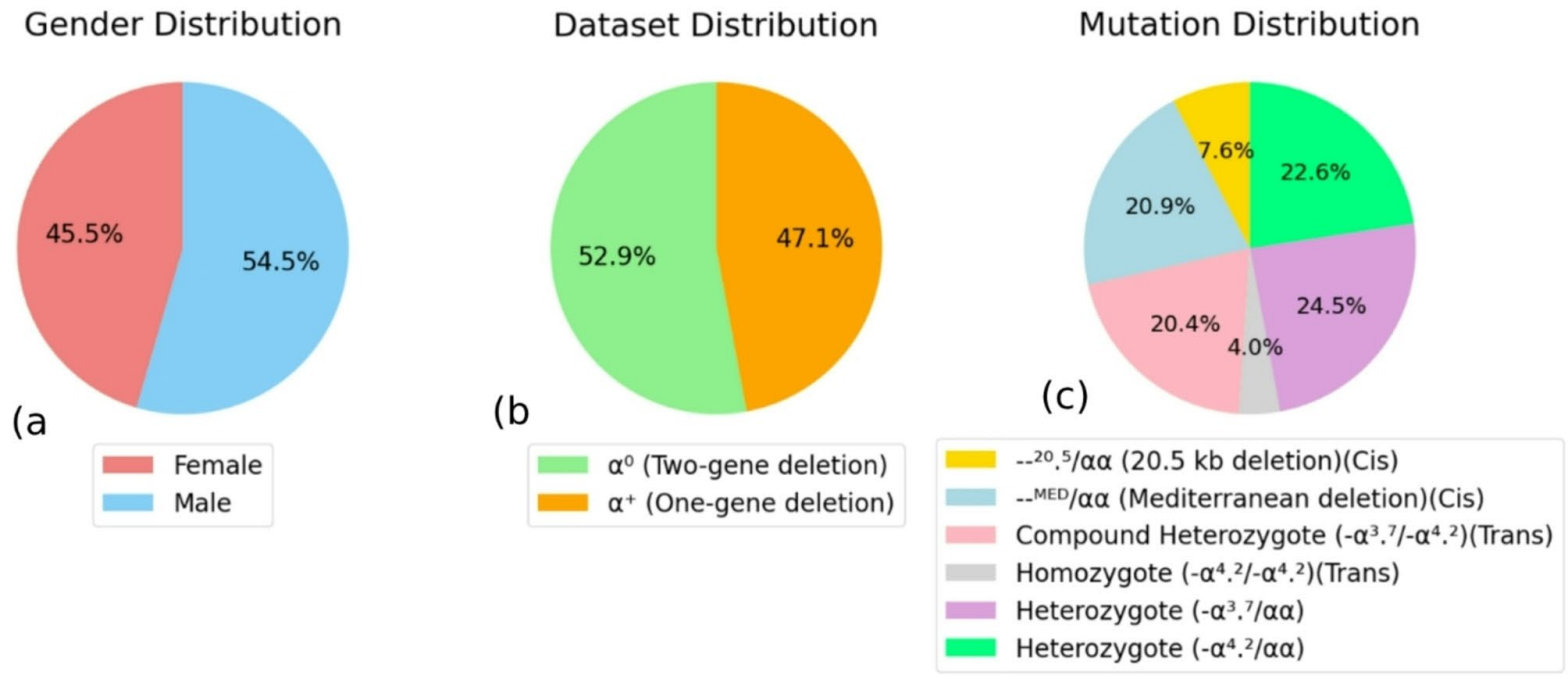
Data distribution in alpha-thalassemia dataset.

The dataset included 956 patients, consisting of 435 females and 521 males (Fig. 2a). Of these, 506 were diagnosed with α⁰ thalassemia (two-gene deletions) and 450 with α⁺ thalassemia (single-gene deletion) (Fig. 2b). The α⁺ group comprised 234 cases of heterozygous α ^3.7,^ 216 cases of heterozygous − α⁴.², 195 cases of compound heterozygous − α³.⁷/−α⁴.², and 38 cases of homozygous − α⁴.² mutations. The α⁰ group included 73 cases of − α²⁰.⁵ and 200 cases of −−Med deletions (Fig. 2c).

- Data timeframe and sample size.

This research was conducted over a period spanning from 2001 to 2023. The study focused on individuals aged 15 to 45 diagnosed with alpha-thalassemia and referred for premarital hematological and genetic screening related to thalassemia. From a larger archive of 20,399 records on hemoglobinopathies, a subset of 1,167 cases with relevant alpha-thalassemia mutations was extracted for analysis. Following screening by a specialist to remove records with co-existing iron deficiency anemia and cases with significant missing data, a total of 956 eligible records were finalized.

- Devices and measurement techniques.

Data were obtained from hospital records, including referral forms from health centers, complete blood count (CBC) results generated using a Sysmex KX-21 hematology analyzer, and genetic test reports. Genetic analyses for common point deletion-type mutations in the alpha- and beta-globin genes were performed using ARMS-PCR, while reverse dot blot techniques were employed in older records. Common large deletions associated with the alpha-globin gene were analyzed using GAP-PCR. Capillary electrophoresis was performed using Sebia and Helena V8 E-class devices, whereas older records were analyzed using cellulose acetate electrophoresis and high-performance liquid chromatography (HPLC). For complex cases, Sanger sequencing was performed using the Applied Biosystems 3130 XL Genetic Analyzer, and advanced deletion detection was carried out using Multiplex Ligation-dependent Probe Amplification (MLPA).

#### Data description

The dataset contained a total of 20 features extracted from the medical records of patients with alpha-thalassemia. Of these, 16 features were related to hematological indices derived from the evaluation of RBCs, white blood cells, and platelets, as measured by CBC analysis. An additional 3 features represented the levels of Hb fractions in the blood, obtained through Hb electrophoresis. Finally, genetic testing was used to identify the type of large deletion mutation present in alpha-thalassemia patients. The details of these features are presented in Table 1.

**Table 1:** Data description.

No.	Data	Description	Unit	Type
1	RBC	Red blood cell count is the number of red cells present per unit volume of blood^26^	×$\:{10}^{6}/$µL	Continuous
2	Hb	Hemoglobin measures the amount of hemoglobin in whole blood^26^	g/dL	Continuous
3	HCT	Hematocrit is the percentage volume of red blood cells in the blood^26^	%	Continuous
4	MCV	Mean corpuscular volume defines the average volume of the red blood cells present^26^	fL	Continuous
5	MCH	Mean corpuscular hemoglobin quantifies the amount of hemoglobin per red blood cell^26^	pg	Continuous
6	MCHC	Mean corpuscular hemoglobin concentrate is the mean hemoglobin concentration per unit volume of red blood cells^26^	g/dL	Continuous
7	RDW	Red cell Distribution width, quantitatively assesses the degree of variation in red cell size^26^	%	Continuous
8	PLT	Platelet count is the number of platelets per unit volume of blood^26^	×$\:{10}^{3}/$µL	Continuous
9	WBC	White blood cell counts or the absolute WBC, is the number of white blood cells present per blood microliter^26^	×$\:{10}^{3}/$µL	Continuous
10	Lym%	Lymphocyte count is the number of Lymphocytes per microliter of blood and is expressed as a percentage of the White blood cell ^26^	%	Continuous
11	MXD%	combines total eosinophils, basophils, and monocytes t in blood circulation^27^	%	Continuous
12	Nuet%	Neutrophil count is the number of neutrophils per microliter of blood and is expressed as a percentage of the White blood cell^26^	%	Continuous
13	Lym#	Absolute lymphocyte count is the number of lymphocytes per microliter of blood^26^	×$\:{10}^{3}/$µL	Continuous
14	PDW	Platelet Distribution Width reflects the size distribution of platelets^28^	fL	Continuous
15	MPV	Mean Platelet Volume describes average size of platelets in the blood^28^	fL	Continuous
16	P-LCR	Platelet larger cell ratio is a percentage of all platelets with a volume measuring over 12 fL circulating in the bloodstream^28^	%	Continuous
17	HbA	Adult hemoglobin^29^ (Hemoglobin A)	%	Continuous
18	HbA2	Hemoglobin A2^29^	%	Continuous
19	HbF	Fetal hemoglobin^29^ (Hemoglobin F)	%	Continuous
20	Gender	Sex of the patient recorded in the medical report	Female = 1 male = 0	Discrete

**Fig. 3:**
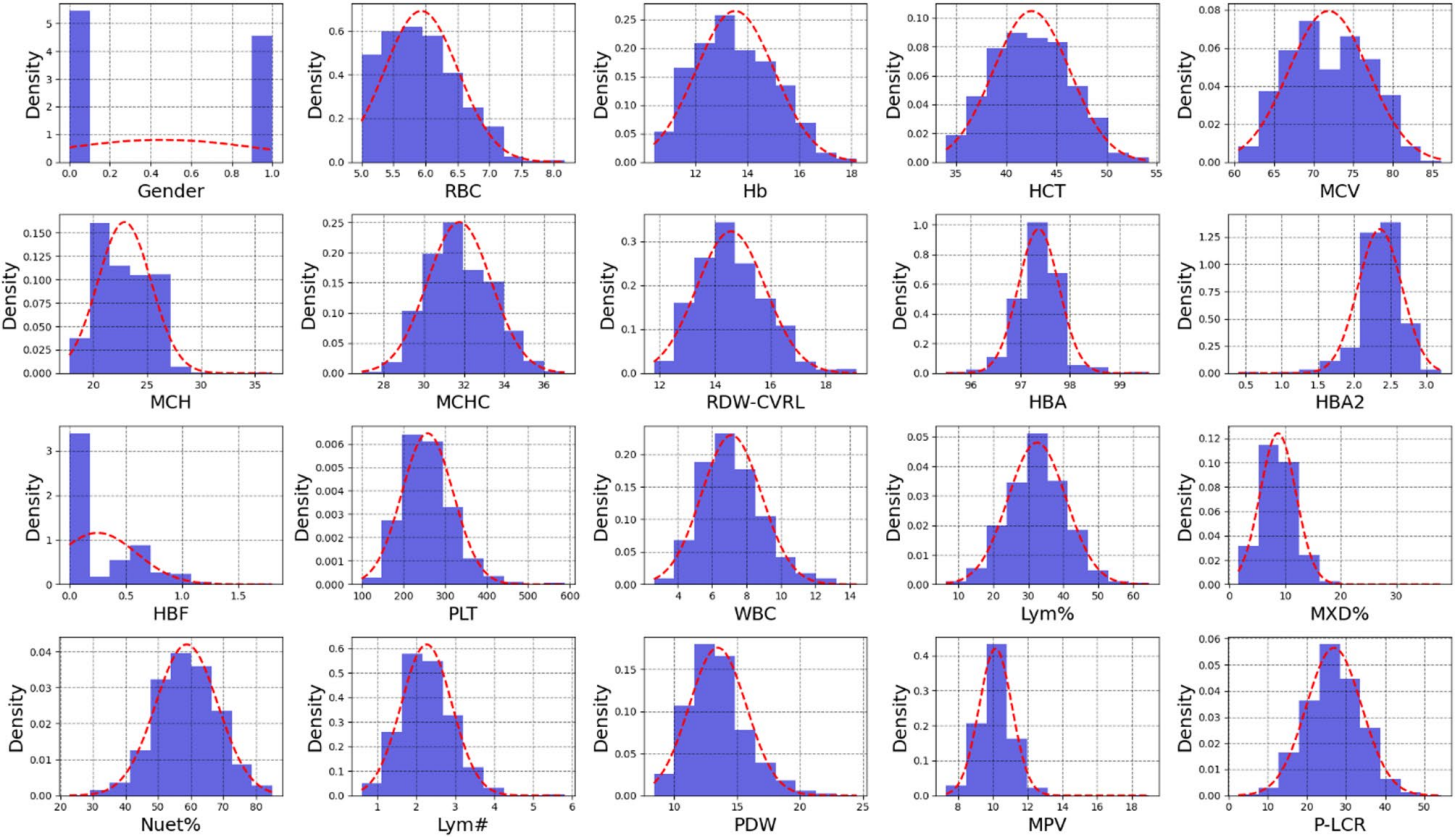
Histograms of hematological parameters in alpha-thalassemia patients.

The distribution of hematological parameters used in this study is demonstrated in Fig. 3. The histograms provide an overview of how each parameter is distributed across the dataset, displaying the variability and range of values for RBC indices, WBC counts, PLT indices, and Hb fractions.

#### Data exploration

- Feature correlation.

**Fig. 4:**
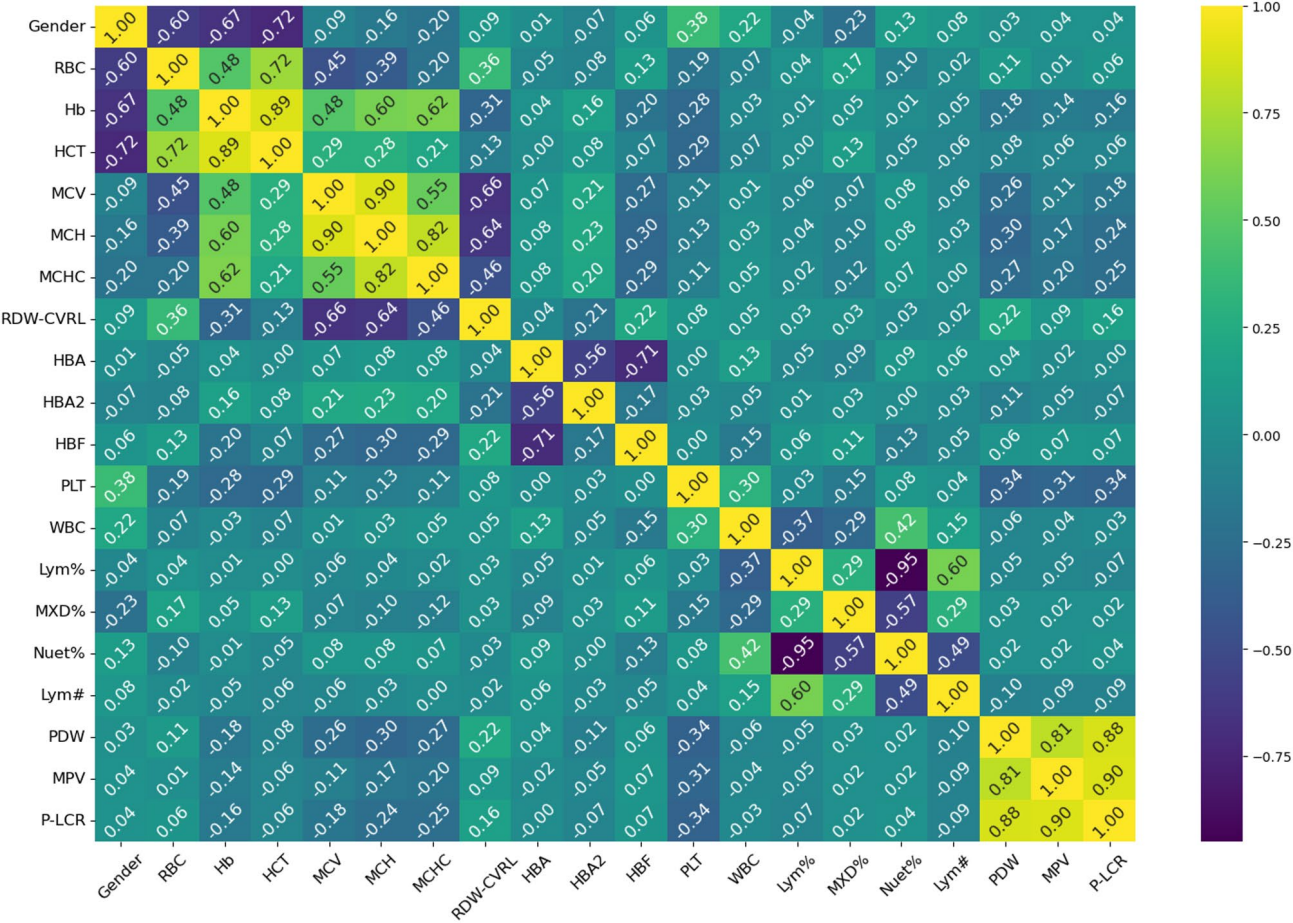
Feature Correlation Matrix of Key Hematological Parameters.

The correlation matrix (Fig. 4) shows several notable relationships. First, Hb and RBC show a moderate positive correlation (0.48), which suggests that an increase in RBC count generally leads to higher Hb levels. In thalassemia, the defective Hb synthesis leads to a reduction in Hb content per RBC which consequently cause the body to compensate for reduced oxygen-carrying capacity by increasing RBC production. However, the correlation is not perfect because these RBCs are microcytic (small size) and hypochromic (pale), meaning they contain less Hb per cell. This results in a moderate positive correlation rather than a strong one. The negative correlation between RBC and MCV (-0.45) aligns with the pathophysiology of α-thalassemia, where increased RBC production compensates for ineffective erythropoiesis, but the newly produced RBCs are smaller in size, leading to lower MCV. Similarly, RBC shows a negative correlation with MCH (-0.39), indicating that as RBC count rises, the Hb content per cell declines, consistent with the microcytic and hypochromic nature of α-thalassemia. However, the weak negative correlation between RBC and MCHC (-0.2) suggests that despite a lower Hb content per cell, the relative concentration of Hb in the smaller RBCs remains somewhat stable, potentially due to a compensatory effect in Hb packing density. The relationships of Hb with MCV (0.48), MCH (0.6), and MCHC (0.62) further highlight these trends. Hb is positively correlated with MCV because larger RBCs typically contain more Hb. The stronger correlation between Hb and MCH (0.6) compared to Hb and MCV suggests that overall Hb content per cell is a more direct determinant of total Hb than cell size alone. The highest correlation, Hb with MCHC (0.62), suggests that the intracellular Hb concentration plays a significant role in determining total Hb levels, despite variations in RBC count and size. The strong correlation between MCV and MCH (0.9) reinforces the concept that larger RBCs tend to contain more Hb, as expected in normal erythropoiesis. The correlation between MCH and MCHC (0.82) further confirms that Hb content per RBC and its concentration within the cell are closely linked. Consequently, the moderate correlation between MCV and MCHC (0.55) indicates that larger RBCs tend to maintain a higher intracellular Hb concentration, though this relationship is not as strong as the MCV_MCH correlation due to variations in Hb synthesis efficiency. By integrating these relationships, the correlation between HCT and RBC (0.72) can be justified. HCT is calculated as [(RBC×MCV)/10]^26, ^meaning that while MCV is reduced in α-thalassemia, the substantial increase in RBC offsets this effect, leading to an overall positive correlation with HCT. The very strong correlation between HCT and Hb (0.89) reflects that total Hb levels and HCT are directly related, both being dependent on RBC count and Hb content per RBC. Examining the RDW, its negative correlation with Hb (-0.31) suggests that greater variability in RBC size (anisocytosis) is associated with lower total Hb. In α-thalassemia, impaired Hb synthesis results in a heterogeneous population of RBCs with varying sizes and Hb content. The body compensates by increasing erythropoiesis, but many newly produced RBCs remain small and hypochromic, contributing to lower total Hb. The weak positive correlation between RDW and RBC (0.36) indicates that increased RBC count is associated with a wider distribution of cell sizes, likely due to the presence of both microcytic and relatively normocytic cells. The strong negative correlations of RDW with MCV (-0.66), MCH (-0.64), and MCHC (-0.46) highlight that greater size variation among RBCs is predominantly due to a higher proportion of smaller, hypochromic cells, reinforcing the hallmark microcytic hypochromic profile of α-thalassemia.

The usual proportion of Hb types in normal adults is 95% to 98% HbA, 2% to 3% HbA2, and < 2% HbF^29^, and the total sums to 100%. Given that HbA levels are largely stable in α-thalassemia carriers (97.45% in α⁺ and 97.31% in α⁰), and since HbA is the dominant fraction (~ 97%), even minor fluctuations in HbA2 and HbF can create noticeable inverse correlations with HbA. The negative correlation between HbA and HbF (-0.7) is justified by the observation that HbF is slightly higher in α⁰ compared to α⁺, suggesting that when HbA slightly decreases, HbF increases to maintain balance. Similarly, the negative correlation between HbA and HbA2 (-0.56) arises because HbA2 is slightly lower in α⁰ than in α⁺. In contrast, the correlation between HbF and HbA2 (-0.27) is weak, suggesting that their variations are largely independent.

Although the mean values for PLT count, MPV, PDW, and P-LCR remain within normal limits in both α⁺ and α⁰ thalassemia, a weak inverse correlation is observed between PLT and MPV (-0.31), PDW (-0.34), and P-LCR (-0.34). This suggests that individuals with relatively higher PLT counts tend to have slightly smaller and less heterogeneous platelets, while those with mildly lower PLT counts exhibit modest increases in MPV, PDW, and P-LCR. However, because these indices lie within the normal range and the correlations are weak, the relationship does not indicate a clinically significant trend. Meanwhile, the strong positive correlations between PDW and MPV (0.8), PDW and P-LCR (0.88), and MPV and P-LCR (0.9) suggest that as PLT size (MPV) increases, both the variation in PLT size (PDW) and the proportion of large platelets (P-LCR) also rise. This is expected, as larger platelets are typically younger and more reactive, contributing to greater size variability and a higher proportion of large platelets in circulation.

WBC subtypes are reported as percentages of the total white cell count, and under normal conditions, these percentages sum to approximately 100%. Typical reference ranges include 50–70% neutrophils, 1–5% eosinophils, 0–1% basophils, 2–10% monocytes, and 20–45% lymphocytes^30^. In this context, the strong negative correlation between Lym% and Nuet% (-0.95) indicates that when neutrophils rise, lymphocytes proportionally decrease. Meanwhile, the moderate negative correlation between the MXD% (which includes monocytes, eosinophils, and basophils) and Nuet% (-0.57) follows a similar pattern, although its effect is less pronounced because these cell types collectively account for a smaller fraction of total white blood cells. Ultimately, because these subpopulations must sum to 100%, any increase in one subset inevitably reduces the relative proportions of the others.

The correlation matrix also revealed a strong association between gender and key RBC parameters, with correlations of 0.6 for RBC count, 0.67 for Hb, and 0.72 for HCT. These differences align with known biological variations, where males typically exhibit higher Hb and HCT levels than females. This disparity is influenced by higher testosterone levels in men, which promote erythropoiesis, as well as genetic differences in the erythropoietin gene and its receptor between sexes^31^.

**Fig. 5:**
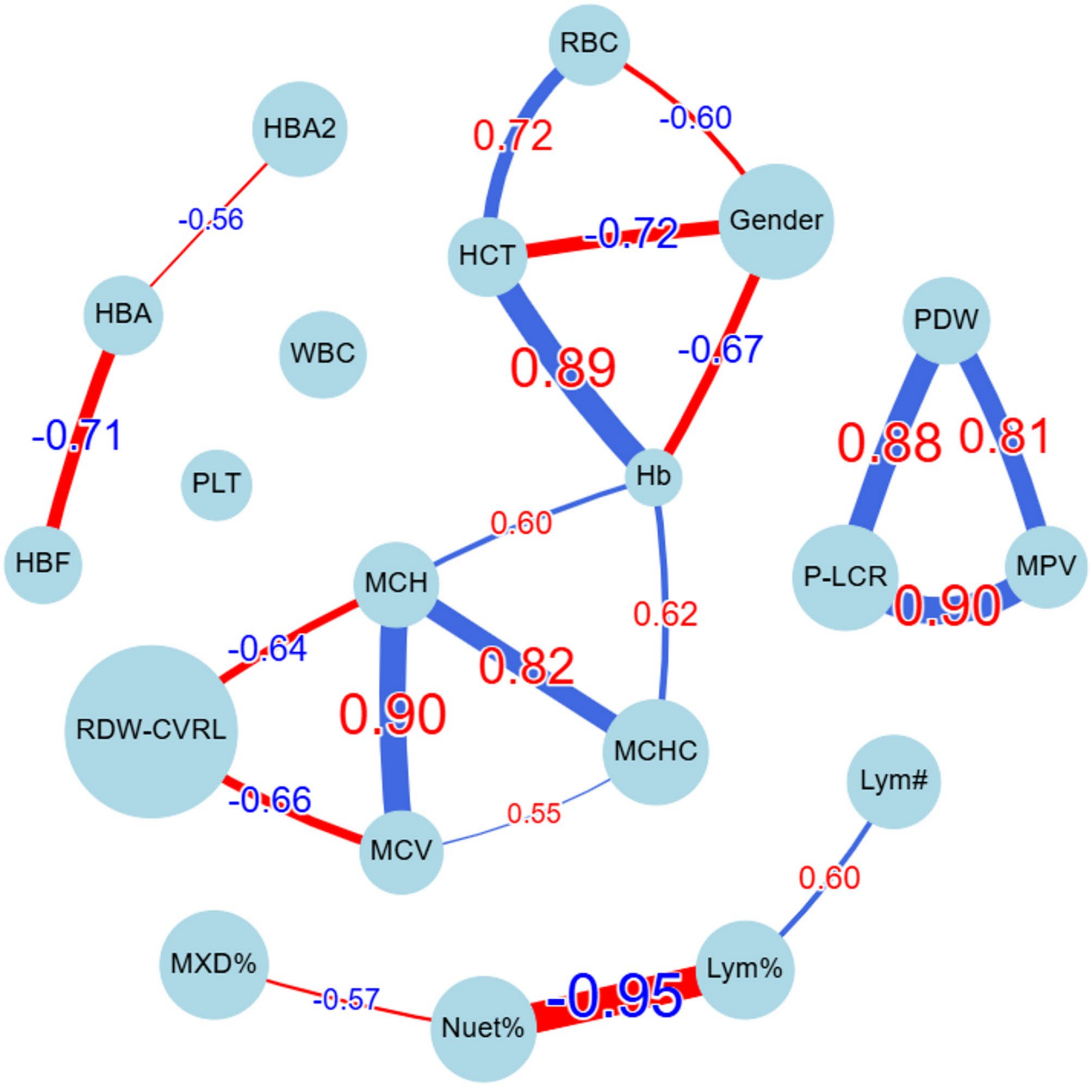
Correlation Network Graph of Blood Parameters in Alpha-Thalassemia Carriers Datasets.

Additionally, Fig. 5, a network graph based on the correlation matrix visualizes relationships between variables. Each node represents a hematological parameter, while the connecting lines indicate correlation strength and direction. Thicker lines show stronger correlations, with red representing negative correlations and blue indicating positive ones.

- Comparative descriptive statistics between α⁺ and α⁰ thalassemia carriers.

The descriptive statistics for both α⁺ and α⁰ thalassemia carriers are summarized in Table 2. Overall, distinct trends were observed between the two groups across various hematological and Hb fractions. In terms of RBC count, both groups showed elevated levels characteristic of α-thalassemia carriers, with α⁰ carriers exhibiting a higher mean RBC count (6.14 ± 0.59 × 10⁶/µL) compared to α⁺ carriers (5.69 ± 0.46 × 10⁶/µL). This reflects the compensatory erythropoiesis common in α⁰ thalassemia, where defective Hb synthesis leads to increased RBC production. The Hb levels were lower in α⁰ carriers (12.96 ± 1.34 g/dL) compared to α⁺ carriers (14.15 ± 1.44 g/dL), matching the more severe anemia in α⁰ thalassemia. Similarly, HCT levels followed this trend, being slightly lower in α⁰ carriers (42.10 ± 3.97%) than in α⁺ carriers (42.98 ± 3.56%). Despite higher RBC counts, this reduction is likely driven by the smaller size of RBCs in α⁰ individuals. The MCV and MCH values were notably reduced in α⁰ carriers (68.61 ± 3.29 fL and 21.12 ± 1.28 pg, respectively) compared to α⁺ carriers (75.56 ± 3.99 fL and 24.92 ± 1.86 pg, respectively), reflecting the more pronounced microcytic and hypochromic features of α⁰ thalassemia. MCHC was also slightly lower in α⁰ (30.77 ± 1.08 g/dL) than in α⁺ (32.89 ± 1.31 g/dL), indicating reduced Hb concentration per unit of red cell volume. RDW was elevated in α⁰ (15.14 ± 1.16%) relative to α⁺ (13.94 ± 1.01%), consistent with increased anisocytosis, a hallmark of α⁰ thalassemia.

For Hb fractions, HbA showed similar means in both groups (~ 97%), though slightly lower in α⁰ (97.31%) than α⁺ (97.45%). HbA2 levels were slightly reduced in α⁰ (2.30 ± 0.30%) compared to α⁺ (2.42 ± 0.29%), while HbF was mildly elevated in α⁰ (0.41 ± 0.35%) relative to α⁺ (0.13 ± 0.29%), which is in line with previous observations of increased HbF in α⁰ thalassemia due to erythropoietic stress^32^.

PLT indices, including PLT, PDW, MPV, and P-LCR, remained within normal ranges in both groups. However, α⁰ carriers displayed slightly higher mean values for PDW (13.96 ± 2.47%) and P-LCR (28.26 ± 6.75%) compared to α⁺ carriers.

For leukocyte indices, WBC counts were similar between α⁰ (6.95 ± 1.74 × 10³/µL) and α⁺ (7.31 ± 1.73 × 10³/µL) groups. Nuet% and Lym% showed comparable distributions, with no major clinical differences between groups, though α⁰ carriers exhibited slightly higher MXD% (9.13 ± 3.56%) than α⁺ (8.14 ± 2.97%).

**Table 2:** Descriptive statistics of blood parameters.

Attributes	Min (α+)	Max (α+)	Mean (α+)	Std Dev (α+)	Min (α0)	Max (α0)	Mean (α0)	Std dev (α0)
RBC (×$\:{10}^{6}/$µL)	5.000	7.160	5.695	0.461	5.000	8.170	6.145	0.587
Hb (g/dL)	10.500	18.200	14.153	1.441	10.400	17.100	12.963	1.338
HCT (%)	34.600	54.100	42.981	3.558	34.000	54.200	42.104	3.972
MCV (fL)	62.500	86.100	75.561	3.994	60.500	77.800	68.612	3.286
MCH (pg)	18.900	36.600	24.918	1.864	17.800	25.500	21.116	1.283
MCHC (g/dL)	29.100	37.000	32.885	1.311	26.900	35.000	30.773	1.079
RDW-CVRL (%)	11.800	18.800	13.938	1.013	12.300	19.110	15.138	1.159
HBA (×$\:{10}^{3}/$µL)	95.500	98.600	97.447	0.392	95.700	99.600	97.307	0.439
HBA2 (×$\:{10}^{3}/$µL)	1.100	3.200	2.419	0.290	0.400	3.200	2.295	0.300
HBF (%)	0.000	1.800	0.133	0.286	0.000	1.800	0.415	0.354
PLT (%)	99.000	584.000	256.444	61.494	113.000	587.000	258.504	62.317
PDW (%)	8.400	21.600	12.841	2.172	8.900	24.500	13.955	2.472
MPV (×$\:{10}^{3}/$µL)	7.300	12.700	9.941	0.989	7.700	19.000	10.256	0.959
P-LCR (fL)	3.900	46.200	25.231	7.578	2.500	54.300	28.261	6.746
WBC (fL)	3.700	13.200	7.313	1.728	2.600	14.400	6.946	1.737
Lym (%)	6.500	60.100	32.215	8.183	9.200	64.500	32.652	8.507
MXD (%)	1.700	25.300	8.145	2.967	1.600	38.000	9.135	3.564
Nuet (%)	32.400	85.000	59.532	9.310	22.800	84.100	58.075	9.980
Lym# (%)	0.700	5.500	2.295	0.625	0.600	5.800	2.227	0.661

- Inferential statistics of differences in blood indices between α⁰ and α⁺ carriers.

To evaluate differences in hematological and electrophoresis parameters between individuals with α⁰-thalassemia and α⁺-thalassemia, we conducted a series of statistical comparisons. Following normality assessment using the D’Agostino–Pearson omnibus test, appropriate statistical tests were selected for each feature. For normally distributed variables with equal variances (assessed via Levene’s test), Student’s t-test was applied; for those with unequal variances, Welch’s t-test was used. Non-normally distributed features were compared using the Mann–Whitney U test. To account for the risk of false positives due to multiple hypothesis testing, p-values were adjusted using the Benjamini–Hochberg false discovery rate correction. Effect sizes (Cliff’s delta, δ) were calculated for each comparison and classified according to established benchmarks: negligible (< 0.15), small (0.15–0.33), medium (0.33–0.47), and large (≥ 0.47).

Out of the 19 evaluated hematologic features, 16 demonstrated statistically significant differences (adjusted *p* < 0.05) between α⁰- and α⁺-thalassemia carriers. Several parameters, including MCV, MCH, MCHC, and RDW-CVRL, demonstrated large effects (δ ≥ 0.47), indicating marked phenotypic separation and robust discriminative potential between the two genotypic groups. Additional parameters such as RBC, Hb, and HbF showed medium effects (0.33 ≤ δ < 0.47) with strong statistical significance, further supporting their role in differentiating α-thalassemia subtypes. Markers such as HBA, HBA₂, PDW, MPV, and P-LCR displayed small effects (0.15 ≤ δ < 0.33), suggesting modest but potentially relevant contributions to the phenotype.

In contrast, PLT, Lym%, and Nuet% showed no significant group differences (adjusted *p* > 0.05) and negligible effect sizes, indicating minimal diagnostic utility. Finally, WBC and MXD%, as well as Lym# were statistically significant but yielded negligible effect sizes (δ < 0.15), suggesting that while differences are detectable statistically, they may not translate into clinical relevance.

Taken together, these findings highlight a subset of hematologic indices, particularly red cell parameters and globin fractions as the most informative for distinguishing between α⁰- and α⁺-thalassemia carriers, with implications for both diagnostic strategies and the development of predictive models in clinical settings.

### Data preparation

The dataset underwent a series of preprocessing steps to ensure data quality and suitability for machine learning analysis. The process began with dataset annotation by labeling each sample as either $\:{\alpha\:}^{+}$ or $\:{\alpha\:}^{0}$, in which six common large mutations associated with alpha-thalassemia were categorized into two classes: alpha-plus ($\:{\alpha\:}^{+}$) and alpha-zero ($\:{\alpha\:}^{0}$). Patients with heterozygous α^3.7^ or α^4.2^ mutations were labeled as alpha-plus, whereas those with − α^20.5^, −−^Med^, compound heterozygous α^3.7^/α^4.2^, and homozygous α^4.2^ were categorized as alpha-zero. The gender variable, as a categorical feature, was encoded using one-hot encoding to convert it into binary form for compatibility with machine learning models. Subsequently, irrelevant features such as Medical Record Number (MRN) were removed, as they lacked analytical significance, to enhance model efficiency and reduce potential noise. Next, data cleaning was performed to address common errors introduced during the manual transfer of data from paper-based medical records to a CSV file. Errors such as typographical mistakes, column or row shifting, format inconsistencies, and duplicate records, which occurred due to patients being referred to the center multiple times over the 22-year period, were detected and rectified to ensure data integrity. These preprocessing steps eliminated noise, irrelevant features, addressed data incompleteness, and optimized the dataset for subsequent machine learning applications.

#### Handling missing value

A critical aspect of data preprocessing involved handling missing values to prevent biases and inaccuracies in the analysis. For continuous features, including Hb, RDW-CVRL, HBA, Platelet, WBC, Lym%, MXD%, Nuet%, Lym#, MPV, and P-LCR, missing values were imputed using the K-nearest neighbors (KNN) algorithm. This method calculates Euclidean distances to identify the five nearest neighbors and replaces missing values with the average of these neighbors, thereby preserving data distribution patterns and ensuring consistency in data representation.

#### Outliers detection

**Fig. 6:**
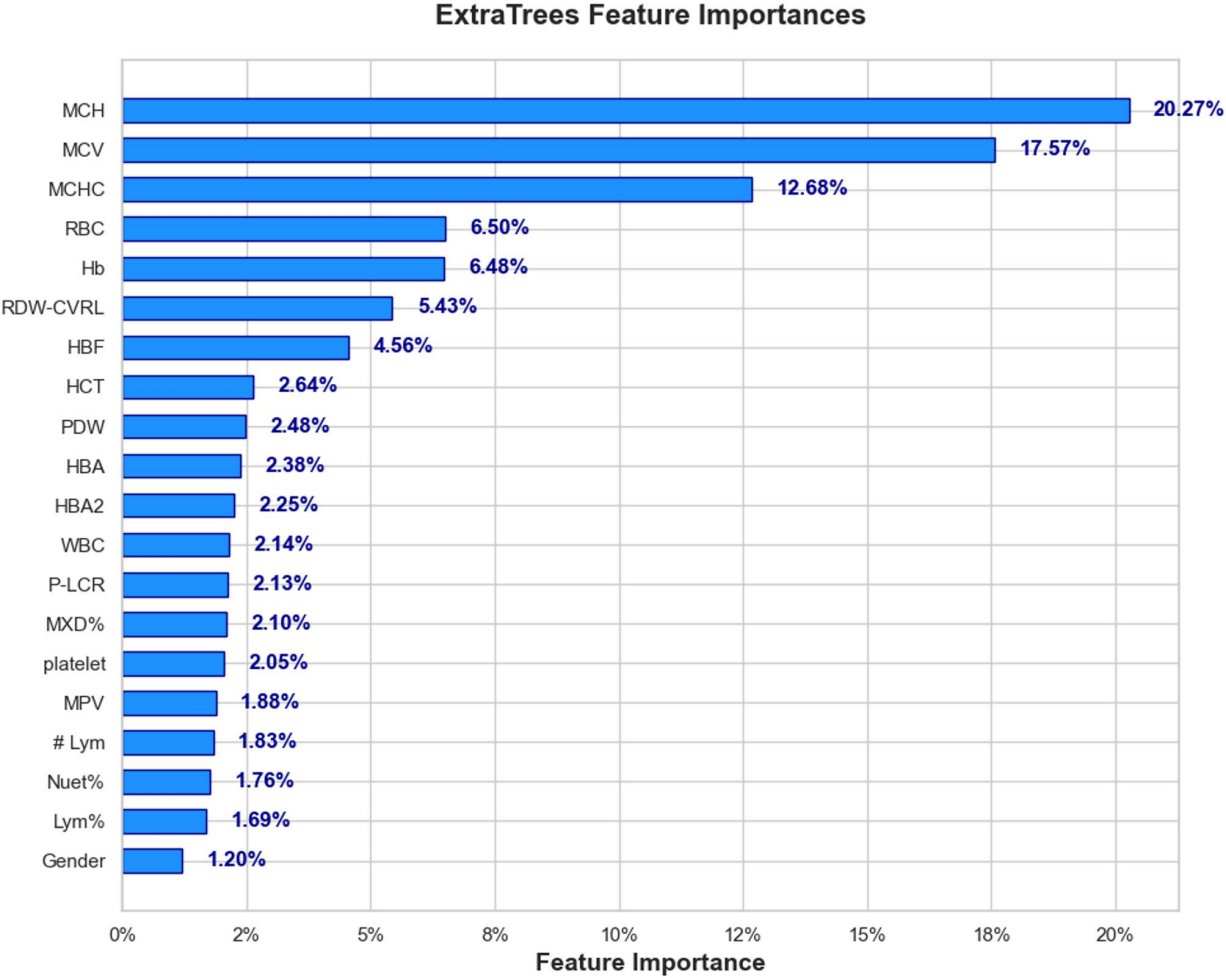
Feature Importance Analysis of Hematological Parameters Using ExtraTreesClassifier.

In this study, the Local Outlier Factor (LOF)^33^ algorithm was employed to identify abnormal samples within the dataset. First, the dataset containing hematological parameters of α-thalassemia carriers was input into the model. The algorithm then computed the distances between each data point and its five nearest neighbors (n_neighbors = 5) using the Euclidean distance metric (*p* = 2). Following this, the local reachability density (LRD) for each sample was calculated based on these distances. The LOF score for each record was then derived by comparing its LRD with those of its neighboring points. Finally, data points with LOF scores exceeding a predefined threshold were marked as outliers and excluded from further modeling. A total of 18 outliers were detected and subsequently removed from the dataset, as their limited presence did not allow for meaningful analysis or reliable insights. This decision ensured that the model remained robust without being influenced by a small set of anomalous cases. This process is consistent with the general LOF methodology described by Zou et al. (2023) (Fig. 6)^34^ (Tables 3, 4).

**Table 3:** Significant differences in hematologic parameters between α⁰- and α⁺-thalassemia groups.

Feature	Test used	Test statistic	Raw *p*-value	Adjusted *p*-value	Effect size (δ)	Impact
RBC	Mann–Whitney U	148,277	< 0.001	< 0.001	–0.443	Medium, significant
Hb	Mann–Whitney U	55,988	< 0.001	< 0.001	0.455	Medium, significant
HCT	Mann–Whitney U	88,135	< 0.001	< 0.001	0.142	Negligible, significant
**MCV**	**Mann–Whitney U**	**20,036**	**< 0.001**	**< 0.001**	**0.805**	**Large**,** significant**
**MCH**	**Mann–Whitney U**	**135,676**	**< 0.001**	**< 0.001**	**0.868**	**Large**,** significant**
**MCHC**	**Mann–Whitney U**	**21,944**	**< 0.001**	**< 0.001**	**0.786**	**Large**,** significant**
**RDW-CVRL**	**Mann–Whitney U**	**163,176**	**< 0.001**	**< 0.001**	**–0.588**	**Large**,** significant**
HBA	Mann–Whitney U	77,426	< 0.001	< 0.001	0.246	Small, significant
HBA2	Mann–Whitney U	70,731	< 0.001	< 0.001	0.312	Small, significant
HBF	Mann–Whitney U	141,238	< 0.001	< 0.001	–0.375	Medium, significant
WBC	Mann–Whitney U	91,093	0.003	0.004	0.113	Negligible, significant
MXD%	Mann–Whitney U	117,849	< 0.001	< 0.001	–0.147	Negligible, significant
Lym#	Mann–Whitney U	93,640	0.021	0.025	0.089	Negligible, significant
PDW	Mann–Whitney U	133,035	< 0.001	< 0.001	–0.295	Small, significant
MPV	Mann–Whitney U	123,906	< 0.001	< 0.001	–0.206	Small, significant
P-LCR	Mann–Whitney U	130,001	< 0.001	< 0.001	–0.265	Small, significant

Adjusted *p*-values were calculated using the Benjamini–Hochberg false discovery rate (FDR) correction.

Significant values are in bold.

**Table 4:** Non-significant hematologic parameters (*p* ≥ 0.05) in α⁰ vs. α⁺ thalassemia comparison.

Feature	Test used	Test statistic	Raw *p*-value	Adjusted *p*-value	Effect size (δ)	Impact
PLT	Mann–Whitney U	104,466	0.663	0.699	–0.017	Negligible, not significant
Lym%	Student’s t-test	0.237	0.813	0.813	0.016	Negligible, not significant
Nuet%	Student’s t-test	–1.481	0.139	0.155	–0.099	Negligible, not significant

Adjusted *p*-values were calculated using the Benjamini–Hochberg false discovery rate (FDR) correction.

#### Feature selection

To improve model performance, feature selection was conducted using the Extra Trees algorithm^35^, an ensemble method that builds multiple randomized decision trees. Unlike Random Forest, which uses bootstrap sampling and selects the best split based on impurity, Extra Trees uses the entire dataset without resampling and chooses split points randomly from a subset of features, reducing variance and improving generalization, especially for high-dimensional or noisy datasets^36,37^. Extra Trees constructs trees using the entire dataset without resampling and applies random splits for feature selection, increasing diversity and reducing variance^37,38^. A key advantage of Extra Trees is its built-in feature selection mechanism, where features are ranked based on their contribution to the reduction of impurity, a measure known as Gini Importance or Mean Decrease in Impurity. Features with higher scores are considered more relevant for the classification task, while those with lower importance can be discarded. This ranking process helps in dimensionality reduction, improving computational efficiency and preventing overfitting. The top-k most important features are often selected based on their ranking, ensuring that only the most relevant features are retained for model training^38,39^.

The process of tree construction in Extra Trees follows a systematic yet randomized approach. At each node, a random subset of features is selected, and multiple potential split points are randomly determined. The best split is then chosen from these randomly generated options, which helps prevent overfitting and enhances the model’s generalization ability. The splitting decision is typically based on an impurity criterion such as Gini Index or Entropy, ensuring an effective division of the dataset into homogeneous subgroups. This process continues recursively until a stopping criterion, such as a minimum number of samples per leaf node, is met^40,41^ Extra Trees Classifier provides several advantages over other tree-based methods. By incorporating randomness in split selection, it exhibits lower variance and is less prone to overfitting compared to models that select the best split deterministically. Additionally, its computational efficiency is higher than that of Random Forest since it does not perform an exhaustive search for the optimal split, making it faster when handling large datasets. Moreover, the ensemble nature of the model, where predictions are aggregated through majority voting for classification or averaging for regression, enhances overall stability and accuracy^39,42^. The blood parameters were evaluated through this approach to identify the most informative features for alpha-thalassemia classification. The performance of the Extra Trees algorithm is influenced by several hyperparameters that control tree growth, feature selection, and prediction stability. These hyperparameters, along with their respective values, are summarized in Table 5.

**Table 5:** Hyperparameters of extra trees feature selection.

Hyperparameter	Value	Explanation
n_estimators	100	Provides a balance between computational efficiency and predictive performance.
max_features	“sqrt”	Enhances diversity among trees by considering a subset of features per split.
max_depth	None	Allows unrestricted tree growth, with pruning applied based on validation performance to prevent overfitting.
min_samples_split	2	Ensures flexibility in learning patterns by setting the minimum number of samples required to split a node.
min_samples_leaf	1	Allows flexibility in learning by setting the minimum number of samples per leaf.
bootstrap	False	Unlike Random Forest, no bootstrap sampling is used, allowing each tree to train on the entire dataset.
criterion	“gini”	Uses the Gini impurity to evaluate the quality of splits, measuring class distribution heterogeneity.
random_state	42	Ensures reproducibility by fixing the random state for consistency across runs.

###  Modeling

#### General experimental framework

All four algorithms: Logistic Regression (LR), Support Vector Machines (SVM), KNN, and Gradient Boosting (GB) were implemented within a standardized scikit-learn pipeline incorporating *z*-score normalization via StandardScaler to ensure consistent feature scaling. Model development followed a nested stratified cross-validation (CV) strategy with an outer 10-fold CV for unbiased generalization estimation and an inner 5-fold CV for hyperparameter tuning via GridSearchCV. This procedure was repeated across five random seeds (42, 123, 456, 789, 2023) to minimize variance from data partitioning. Class imbalance between α⁺ and α⁰ carriers was addressed using class_weight=’balanced’ where applicable. Model selection within the inner loop prioritized the F1-score, balancing false positives and false negatives to reflect the clinical importance of both error types.

Following hyperparameter optimization,the best model from each outer fold underwent probability calibration using CalibratedClassifierCV (5-fold, training data only) to improve clinical interpretability of predicted risk scores. Sigmoid (Platt scaling) or isotonic regression was applied depending on model suitability. Performance was evaluated at a fixed 0.5 threshold using six metrics: accuracy, precision, recall, specificity, F1-score, and AUC-ROC (Receiver Operating Characteristic Area Under the Curve) with results reported as mean ± standard deviation across all folds and seeds. The final version of each model was retrained on the full dataset using the optimal hyperparameters and saved for reproducibility.

#### Model-specific details

- LR:
- Explored three regularization schemes: L1 (liblinear solver), L2 (lbfgs or saga solvers), and Elastic Net (saga solver) with l1_ratio ∈ {0.1, 0.5, 0.9}. The inverse regularization strength C spanned 10⁻⁴ to 10⁴ (logarithmic scale). The maximum number of iterations was set to 5000 to ensure convergence.
- SVM:
- Tested both linear and RBF kernels, with C ∈ {0.01, 0.1, 1, 10, 100}. For RBF, gamma ∈ {0.001, 0.01, 0.1, 1, ‘scale’, ‘auto’}. Probability estimation was enabled (probability = True) to support calibration and decision-threshold adjustment.
- KNN:
- Used distance-weighted voting (weights=’distance’) with n_neighbors ranging from 3 to 30 (odd integers), p ∈ {1, 2} for Manhattan/Euclidean metrics, and leaf_size ∈ {10, 20, 30, 50}. Search algorithms included {‘auto’, ‘ball_tree’, ‘kd_tree’, ‘brute’}.
- GB:

Hyperparameter search included n_estimators ∈ {50, 100, 200}, learning_rate ∈ {0.01, 0.05, 0.1}, max_depth ∈ {3, 4, 5}, min_samples_split ∈ {2, 5, 10}, min_samples_leaf ∈ {1, 2, 4}, subsample ∈ {0.8, 0.9, 1.0}, max_features ∈ {‘sqrt’, ‘log2’, None}, and ccp_alpha ∈ {0, 0.001, 0.01}.

#### Stacking ensemble

The stacking ensemble was constructed to leverage the complementary strengths of four independently optimized base classifiers: GB, SVM, KNN, and LR, whose hyperparameters were fixed at the optimal values identified in prior single-model experiments.

Configurations were:

- GB: learning_rate = 0.05, max_depth = 5, min_samples_leaf = 4, min_samples_split = 2, subsample = 0.8, n_estimators = 200, max_features = None, ccp_alpha = 0, random_state = 42.
- SVM (RBF): C = 100, gamma = 0.001, kernel=’rbf’, probability = True, class_weight={0:w0,1:w1}, random_state = 42 within a StandardScaler() pipeline.
- KNN: n_neighbors = 19, p = 1, weights=’distance’, leaf_size = 10, algorithm=’auto’, metric=’minkowski’ within a StandardScaler() pipeline.
- LR (elastic net): C = 0.01, l1_ratio = 0.1, penalty=’elasticnet’, solver=’saga’, max_iter = 5000, class_weight={0:w0,1:w1}, random_state = 42 within a StandardScaler() pipeline.

Class imbalance between α⁺ and α⁰ carriers was handled in applicable models via class_weight derived from training-set class frequencies. The meta-learner was a LR model embedded in a StandardScaler() pipeline. Hyperparameters were optimized via grid search with 5-fold CV on out-of-fold meta-features using the search space:

- penalty ∈ {‘l1’,‘l2’,‘elasticnet’}, C ∈ np.logspace(-4,4,9), solver ∈ {‘liblinear’,‘lbfgs’,‘saga’}, l1_ratio ∈ {0.1,0.5,0.9} (elastic net only), and class_weight ∈ {None,‘balanced’}; max_iter = 5000, random_state = 42.

To prevent information leakage into meta-training, meta-features were the base learners’ out-of-fold predicted probabilities computed with 5-fold CV on each outer-fold training partition. Model selection followed a nested stratified CV with an outer 10-fold (n_splits = 10, shuffle = True, random_state = 42). Within each outer training fold, base model out-of-fold predicted probabilities were first generated; the meta-learner was then tuned in an inner 5-fold CV with scoring=’f1’. The best meta-learner was integrated into a StackingClassifier(cv = 5, passthrough = False), and the ensemble’s probability outputs were calibrated using a sigmoid method (CalibratedClassifierCV(method=’sigmoid’, cv = 5)), fit only on the training partition of each outer fold.

For decision-making, metrics were reported at the default threshold (0.5) and at a per-fold optimized threshold chosen from the outer test fold precision–recall curve to maximize F1. This threshold selection does not affect model fitting, but uses test labels for selection; therefore, the 0.5-threshold results are treated as the primary unbiased estimates, while the optimized-threshold results are provided as complementary operating points. Performance on outer test folds includes accuracy, precision, recall, specificity, F1-score, and AUC-ROC (AUC is threshold-independent), summarized as mean ± standard deviation across outer folds. Finally, the calibrated stacking model was refit on the full dataset and serialized for reproducibility.

### Evaluation

#### Performance metrics

The performance of all models in classifying α⁰ versus α⁺ thalassemia was assessed using multiple complementary metrics to ensure both statistical robustness and clinical reliability.

- Accuracy quantified the proportion of correctly classified cases overall:

1 $$\:\text{Accuracy}=\frac{TP+TN}{TP+TN+FP+FN}\times\:100$$

While informative, accuracy alone can be misleading for imbalanced data, and therefore was interpreted alongside other metrics.

- Precision measured the proportion of predicted α⁰ cases that were truly α⁰:

2 $$\:\text{Precision}=\frac{TP}{TP+FP}\times\:100$$

High precision reduces false positives (α⁺ misclassified as α⁰), minimizing unnecessary genetic testing and patient anxiety.

- Recall (Sensitivity) quantified the ability to detect true α⁰ cases:

3 $$\:\text{Recall}=\frac{TP}{TP+FN}\times\:100$$

- F1-score, the harmonic mean of precision and recall, balanced these two metrics:

4 $$\:F1=2\times\:\frac{\text{Precision}\times\:\text{Recall}}{\text{Precision}+\text{Recall}}\times\:100$$

This is especially relevant given the trade-off between precision and recall, and the greater clinical harm of false negatives (α⁰ misclassified as α⁺) compared to false positives.

- AUC (Area Under the ROC Curve) provided a threshold-independent measure of discriminative ability, summarizing the trade-off between true positive rate and false positive rate across all decision thresholds.
- Here, the classification outcomes can be summarized in Table 6.

**Table 6:** Classification outcomes in α-thalassemia prediction.

Prediction outcome	Description	Implication
True positives (TP)	α0 cases correctly predicted as α0	The model accurately identifies α0 cases, ensuring proper genetic counseling and follow-up.
True negatives (TN)	α + cases correctly predicted as α+	The model correctly excludes α0 cases, preventing unnecessary genetic testing.
False positives (FP)	α + cases incorrectly predicted as α0	Leads to unnecessary genetic testing, anxiety, and increased medical costs.
False negatives (FN)	α0 cases incorrectly predicted as α+	Risk of missing α0 cases, which may impact premarital counseling and prenatal diagnosis, potentially leading to thalassemia major births.

## Results

### Feature selection result

The present study employed an ExtraTreesClassifier to evaluate the relative importance of various hematological parameters for classification. As shown in the feature importance chart (Fig. 6), RBC indices, particularly MCH, MCV, and MCHC, emerged as the most influential predictors, suggesting that mean Hb content, cell volume, and Hb concentration play central roles in distinguishing between classes. Additional RBC-related measures, such as RBC count and Hb, also ranked highly, further underscoring the prominence of RBC characteristics in the classification task. In contrast, WBC and PLT measures demonstrated moderate contributions, indicating their supportive but less dominant role in predicting the target outcome. Finally, gender was identified as the least influential feature, suggesting minimal influence of demographic factors relative to the comprehensive panel of hematological indices examined.

Based on the feature selection results, a threshold of 2% feature importance was considered. Features with an importance ≥ 2% (e.g., MCH, MCV, MCHC, RBC, Hb, etc.) can be retained for model training, while those below this threshold (e.g., Gender, Lym%, Nuet%) may be excluded to reduce dimensionality and improve model efficiency.

Another group of features was also selected to train the model. These features are part of the collected dataset but are also commonly used by experts in clinical settings for decision-making and follow-ups. They are routinely recorded in medical records and paper forms in premarital screening centers. These selected clinical features include MCH, MCV, MCHC, RBC, Hb, HCT, RDW-CVRL, HbA2 ,and HbF. Integrating these features into the model enhances its alignment with real-world diagnostic practices. An important point to highlight is that among these nine selected clinical features, eight ranked highest in importance according to the ExtraTreesClassifier. This highlights their importance in prediction and their alignment with expert-driven diagnostic criteria.

### Model evaluation result

Across both feature domains, all models demonstrated strong predictive performance. Using the feature-driven set, the stacking classifier achieved the highest performance overall, with an accuracy of 93.24%, F1-score of 93.94%, recall of 98.60%, and an AUC of 95.29%. Gradient Boosting ranked second in terms of accuracy (91.03%) and F1-score (91.88%), followed closely by KNN (91.67% F1-score). LR recorded the lowest recall (93.08%) in this group, although its accuracy (90.59%) remained competitive.

When trained on the clinical feature set, stacking again achieved the top overall performance, with an accuracy of 93.13%, an F1-score of 93.79%, recall of 97.99%, and an AUC of 94.75%. The second-highest performance was observed for KNN, which reached an F1-score of 91.90% and recall of 96.26%. LR once more had the lowest recall (93.25%) but maintained a strong accuracy of 90.48%.

Direct comparison between the two feature sets revealed that the feature-driven approach slightly outperformed the clinical set for most models, particularly in recall for Gradient Boosting and KNN. However, differences were minimal for stacking, indicating that it leveraged both domains effectively.

Fig. 10 illustrates the ROC (Receiver Operating Characteristic) curves for various machine learning models applied to the alpha-thalassemia classification task. Across both feature sets, all models demonstrated high discriminative ability (AUC > 0.93). Using the feature-driven set, the Stacking ensemble achieved the highest AUC (0.9499), followed closely by SVM (0.9472) and LR (0.9467), while KNN yielded 0.9432. With the clinical feature set, Stacking again ranked highest (0.9460), marginally outperforming SVM (0.9448) and LR (0.9441), with KNN showing the lowest but still strong performance (0.9397).

**Fig. 7:**
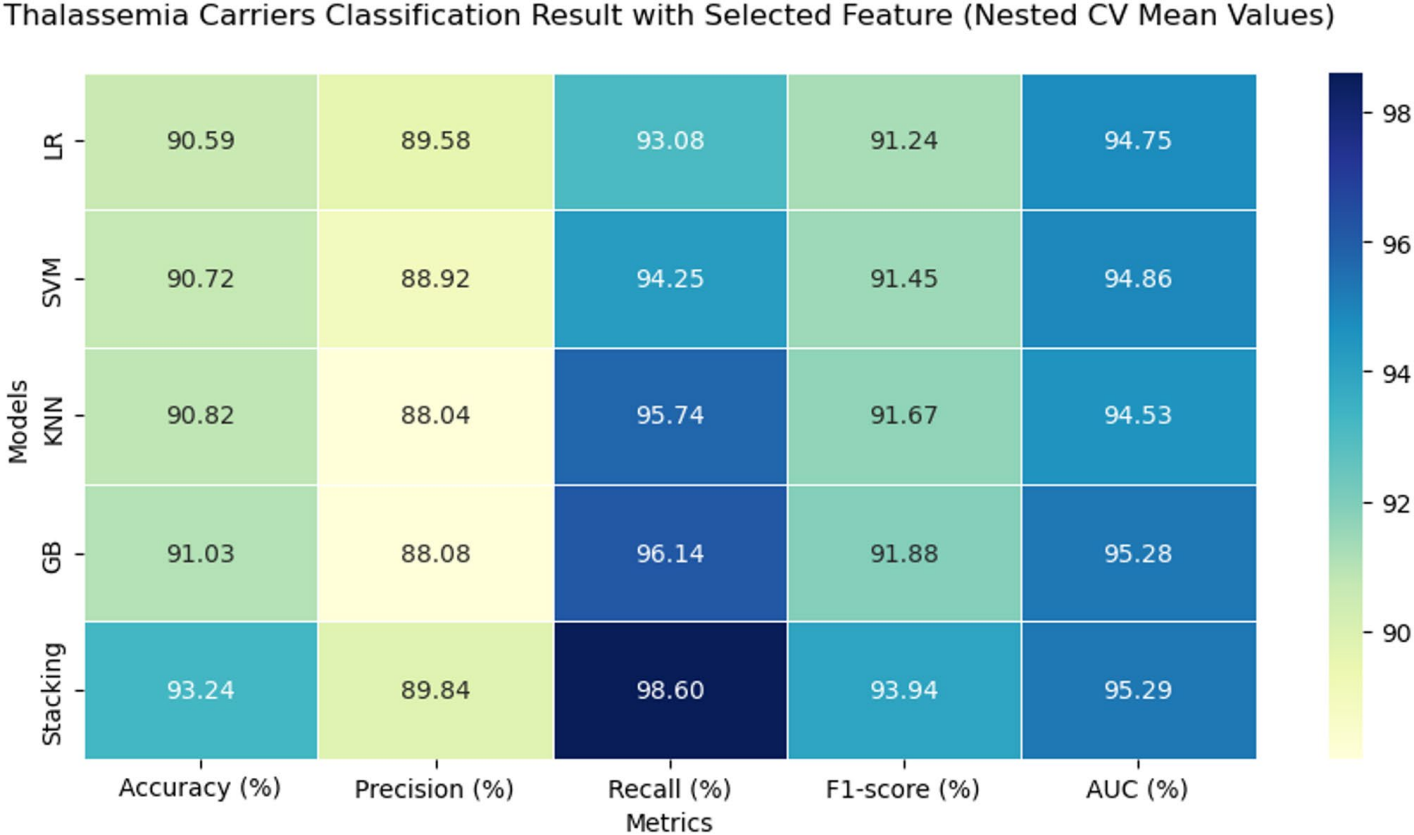
Heatmap for alpha-thalassemia classification (data-driven features).

**Fig. 8:**
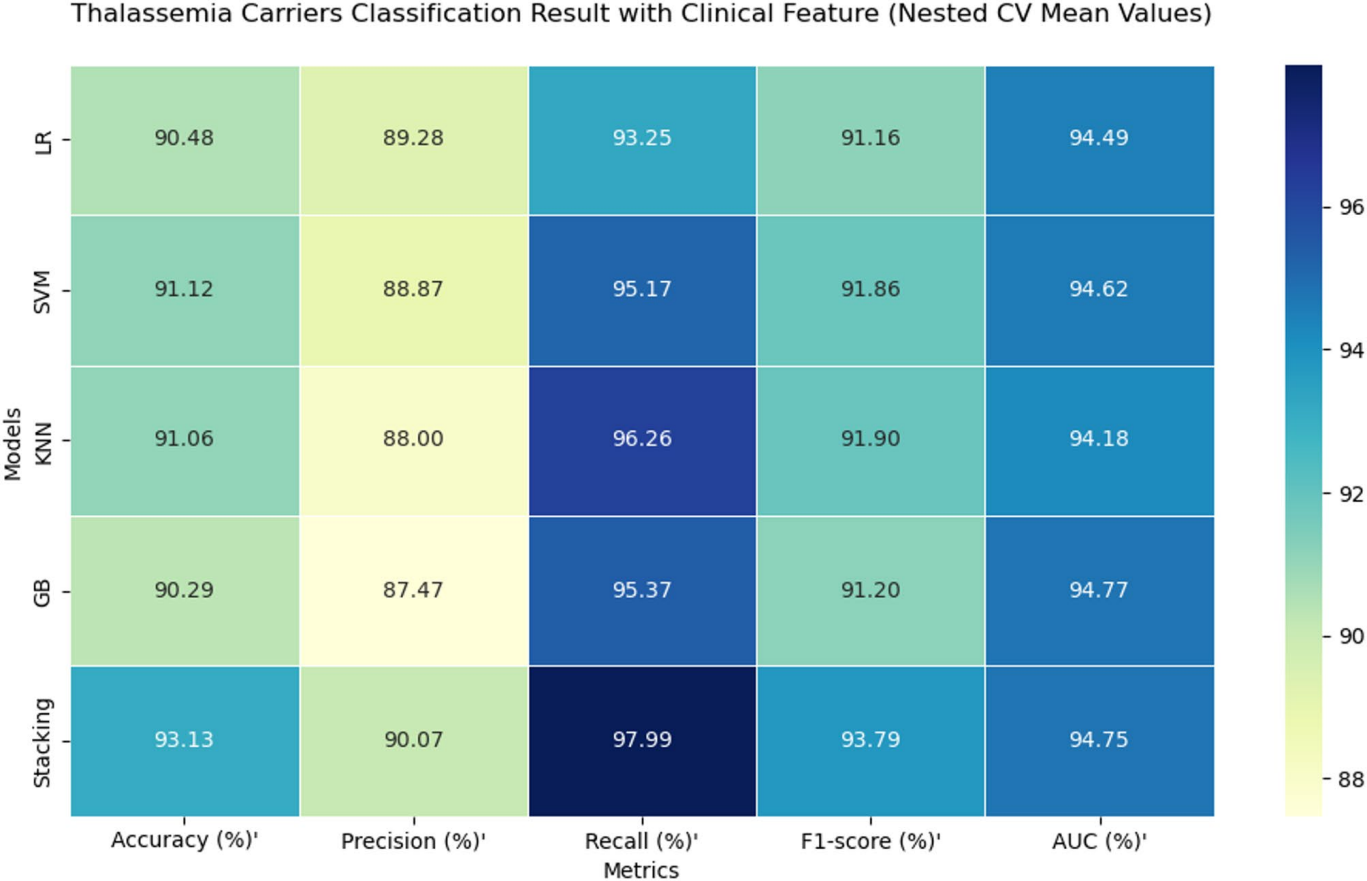
Heatmap for alpha-thalassemia classification (clinical features).

It is important to note that the AUC values reported in the overall performance metrics tables (Figs. 7 and 8) represent the mean across folds and random seeds, whereas the AUC values from the ROC curves (Fig. 10) were obtained by pooling the predicted probabilities from all outer folds. This pooling approach ensures that each prediction used to generate the ROC curve was made on unseen data, providing an unbiased visualization of model discrimination.

## Discussion

**Fig. 9:**
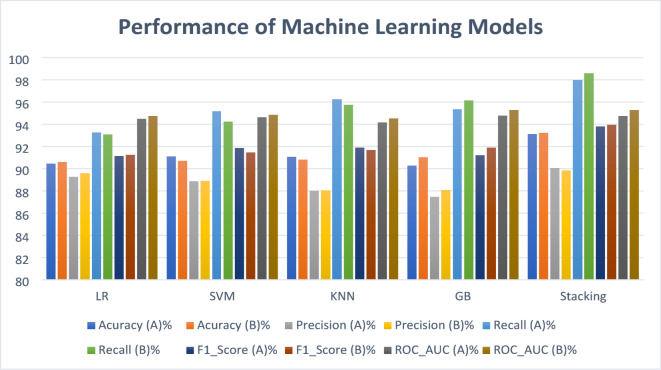
Performance comparison of classifiers based on feature-driven (**A**) and clinical feature (B) feature set.

The results from the correlation matrix (Fig. 4), feature importance analysis using the Extra Trees algorithm (Fig. 6), and statistical testing (Tables 3 and 4) consistently identify the same set of hematological indices as the most discriminative for classifying patients into α⁰-thalassemia and α⁺-thalassemia groups. These features not only exhibited the highest positive or negative correlations with the target classification but also ranked at the top in the Extra Trees importance scores. Importantly, they correspond to hematological indices routinely assessed in clinical settings, further supporting their alignment with expert-driven diagnostic criteria. The statistical tests further confirmed their significance, with large effect sizes indicating a strong and clinically meaningful difference between the two patient groups. This confirms the predictive strength and clinical relevance of these features. Taken together, these findings demonstrate that the most informative features are inter-correlated (Fig. 5), clinically relevant, statistically significant, and consistently prioritized across multiple analytical approaches. This convergence strengthens the reliability of the feature set and supports its utility for predictive modeling of α-thalassemia carrier states in real-world diagnostic workflows. Building on these findings, Fig. 9 shows that the consistent high performance observed across both clinical and feature-driven feature sets among the selected machine learning algorithms suggests that the classification task is inherently well-supported by hematological patterns, regardless of the feature selection strategy.

Our study demonstrates that reducing the input variables from 15 algorithmically selected features to the 9 clinically used features did not result in any meaningful decrease in predictive performance across models. This stability suggests that the clinically curated set captures the core predictive information required for α⁰ vs. α⁺ classification and that classification performance is largely independent of the specific feature set size in this dataset.

By using clinical features, we reduce testing complexity without losing accuracy, making the model more practical for real-world application. Feature selection plays a crucial role in improving machine learning models by reducing dimensionality while preserving predictive performance. Selecting only the most relevant features enhances model interpretability, reduces computational costs, and minimizes unnecessary data collection expenses^43,44^. Additionally, removing redundant or non-informative features helps prevent overfitting, ensuring the model generalizes well to new data^45^. This is particularly important in medical applications, where too much testing can increase patient burden and healthcare costs.

Another critical aspect of this study is handling outliers in α-thalassemia data. The hematological features are influenced by various biological and external factors, such as living at high altitudes^46^, smoking^47^, and underlying diseases like kidney disorders^48^ or hypothyroidism^49^. Additionally, coexisting genetic conditions, such as uncommon point mutations in the globin cluster, further contribute to variability. A key consideration is that HBA2 and HBA1 share the same coding sequence but differ in the introns as well as the 5’ and 3’ UTR, affecting gene expression. For example, HBA2 encodes 2–3 times more protein than HBA1^50^, yet they are typically reported in the same way (e.g., heterozygous 3.7 or heterozygous 4.2) without specifying which gene is affected, potentially influencing hematological parameter interpretations and causing outliers in the data. Outliers may also arise from errors in laboratory procedures or limitations of automated analyzers. For instance, in patients with alpha-thalassemia, the predominance of microcytic RBCs may be misclassified as platelets by automated hematology analyzers due to their small size. This misclassification can result in falsely elevated PLT counts and the erroneous diagnosis of thrombocytosis^51^, introducing misleading data points into the dataset. Using the LOF method, we effectively identified and managed these atypical data points, preventing them from skewing model predictions. This step is essential in medical datasets, where even a few outliers can significantly impact model reliability.

**Fig. 10:**
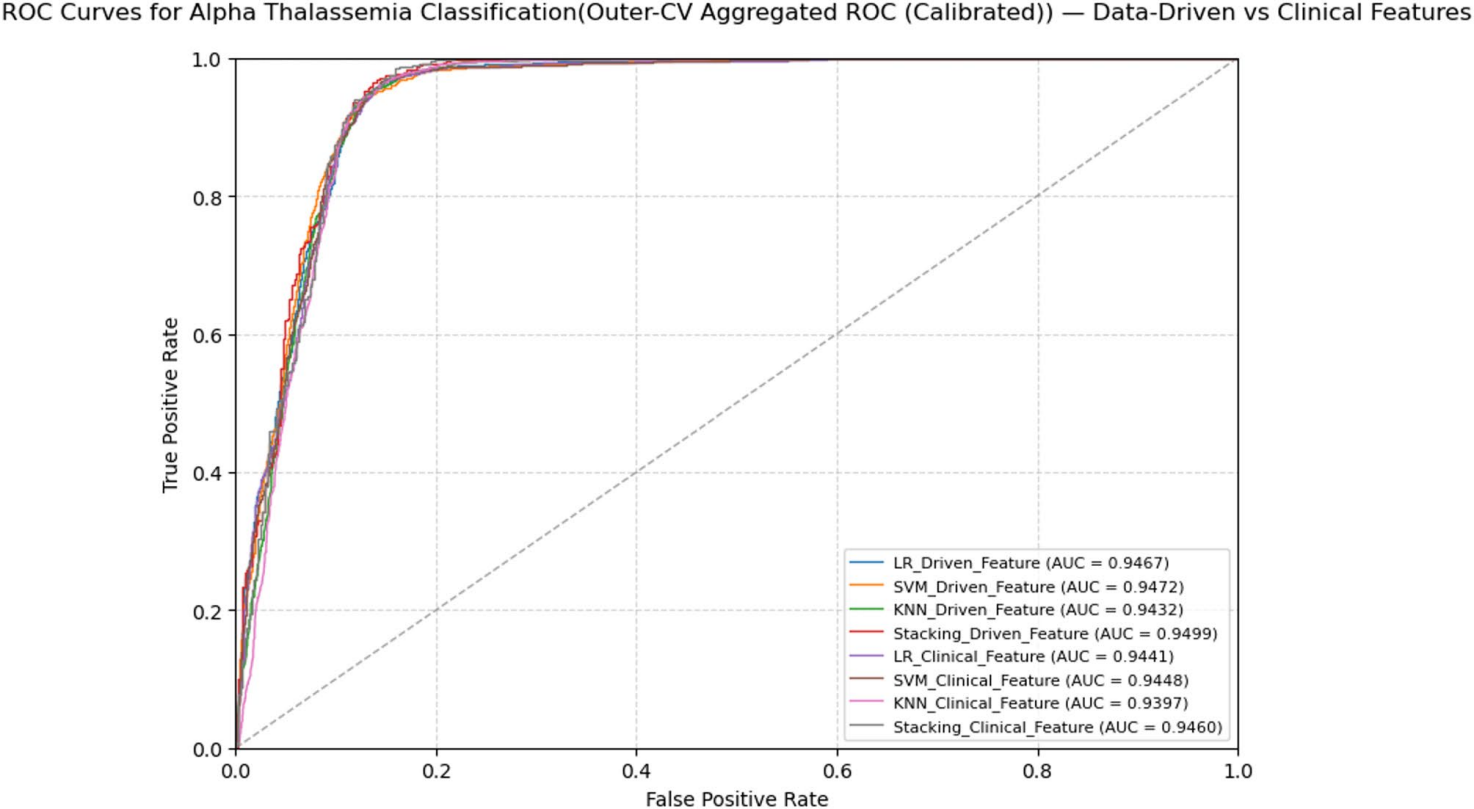
ROC curves for alpha-thalassemia classification.

Among the models evaluated, the stacking ensemble approach proved to have the highest performance across both feature sets. Stacking is particularly effective because it combines multiple base models, each learning different patterns in the data while reducing prediction bias and variance^52,53^. As a result, stacking improves overall accuracy and makes predictions more reliable. A review of 45 studies on ensemble learning for disease prediction (Mahajan et al., 2023)^54^ found that stacking was used in about 50% of cases and had the highest average accuracy (82.6%). In some cases, like liver and diabetes prediction, it even reached 100% accuracy, proving its strength in medical classification tasks (Fig. 10).

Our findings are consistent with those of Phirom et al.^55^in “DeepThal: A Deep Learning-Based Framework for the Large-Scale Prediction of the α+-Thalassemia Trait.” In their study, a deep learning-based framework achieved an accuracy of 80.77%, sensitivity of 70.59%, and AUC of 0.857 using RBC indices. The authors found that MCH was the most critical parameter for predicting α + thalassemia. While their model demonstrated moderate performance, our stacking ensemble approach outperformed it in almost all evaluation metrics, likely due to the combination of multiple models. Similarly, in Feng et al.‘s^56^ study, “An Online Alpha-Thalassemia Carrier Discrimination Model Based on Random Forest,” a random forest model achieved an accuracy of 91.5%, AUC of 0.948, and improved positive predictive value (PPV) by using five clinical features. In comparison, our model achieved higher accuracy and maintained similar AUC values, suggesting that ensemble approaches like stacking can yield superior generalization performance over single tree-based methods.

The findings of Fu et al.^57^in “The TVGH-NYCU Thal-Classifier” are also relevant. Their SVM classifier achieved an AUC of 0.76 and moderate sensitivity and specificity for distinguishing thalassemia from non-thalassemia microcytic anemia. Our study demonstrated significantly higher performance, with an AUC of 95.29% and improved classification error rates, highlighting the advantages of ensemble learning over SVM in complex hematological datasets. In “MultiThal-classifier, a Machine Learning-Based Multi-Class Model for Thalassemia Diagnosis” by Wang et al.^58^, the researchers employed the XGBoost algorithm and SMOTENC for data balancing, achieving sensitivity of 90.27%, specificity of 97.87%, and AUC of 94.07%. Similar to our results, they identified MCV, MCH, and RDW_CVRL as the most influential features. This corroborates our findings that RBC indices are crucial for accurate classification.

Meti et al.^59^, in their work “Advancing Alpha-Thalassemia Carrier Screening for Better Predictions Using Explainable AI,” demonstrated that decision trees and boosting algorithms could achieve accuracy rates of 87%–90.11%. They emphasized the importance of model interpretability using SHAP and LIME. Our stacking model surpassed these results and is similarly positioned for future integration with explainable AI techniques to enhance clinical trust and adoption. Another relevant study, “ThalPred: A Web-Based Prediction Tool for Discriminating Thalassemia Trait and Iron Deficiency Anemia,” by Laengsri et al.^60^, developed an SVM model with a 95.59% accuracy, successfully differentiating thalassemia from other microcytic anemia disorders. Our approach complements their findings by demonstrating the benefits of multi-model integration, resulting in improved precision-recall balance for alpha-thalassemia subtypes. AlAgha et al.^61^, in “Identifying β-Thalassemia Carriers Using a Data Mining Approach,” highlighted the limitations of using complete blood count (CBC) parameters alone for thalassemia diagnosis, emphasizing the need for robust machine learning classifiers. Their naïve Bayesian (NB) model with SMOTE oversampling achieved sensitivity of 98.81% and specificity of 99.47%. Although focused on β-thalassemia, their emphasis on data balancing and comprehensive feature engineering parallels our approach to improving alpha-thalassemia classification.

In summary, our study builds upon these previous works by integrating advanced ensemble learning, optimized feature selection, outlier management, hyperparameter tuning, and comprehensive evaluation metrics. The stacking model’s superior performance highlights the potential for improved thalassemia screening and diagnosis, particularly when combining data-driven and clinically relevant features.

### Limitations

This study demonstrates the effectiveness of machine learning models in classifying alpha-thalassemia subtypes. However, several limitations should be considered.

First, the dataset was sourced from a single region, which may limit the model’s generalizability to other populations with different genetic mutation patterns. Additionally, the dataset size, while substantial (956 samples), may still be insufficient for certain machine learning techniques, particularly deep learning models that typically require larger datasets for optimal performance. Limited access to centralized and continuous data also poses a challenge, restricting the ability to explore broader aspects of the problem. Feature selection relied on the ExtraTrees algorithm, which effectively ranked the most relevant features. However, other potentially useful features may not have been considered due to the reliance on a single selection method. The reduced feature set of nine key parameters improved interpretability but led to a slight drop in model accuracy, suggesting the possible loss of clinically relevant information. Data preprocessing methods also introduce potential biases. The KNN approach was used for imputing missing values, assuming that the nearest neighbors provide an accurate estimate, which may not always hold true. Similarly, the Local Outlier Factor (LOF) technique was used for outlier detection, but it may not capture all types of outliers, potentially excluding valuable data points. Another limitation is the interpretability of machine learning models, particularly ensemble methods, which can be challenging for clinical adoption. The integration of explainable AI techniques, such as SHAP and LIME, is needed to provide clearer insights into model predictions for healthcare professionals.

## Conclusion and future work

This study successfully applied machine learning to classify alpha-thalassemia patients based on the number of deleted genes, achieving high accuracy. Using data from the Thalassemia and Hemophilia Genetics Research Center, we performed preprocessing, selected key features, and evaluated various models. The results showed that clinical models with fewer features achieved accuracy comparable to feature-driven models. This demonstrates their efficiency in reducing diagnostic time, cost, and patient stress, while enhancing decision-making for physicians. Additionally, increasing the number of diagnostic features can impose significant costs on healthcare systems, patients, and healthcare providers. Effective feature selection optimizes model performance while minimizing expenses.

In conclusion, this research highlights that with proper data preprocessing and feature selection, highly accurate and cost-effective models can be developed for classifying alpha-thalassemia, benefiting both clinical practice and healthcare economics.

Future work will focus on expanding the dataset by collecting multi-center data from diverse populations to enhance model robustness and generalizability. This will involve incorporating additional clinical parameters, such as iron profile data , and patient background factors, including smoking status, kidney disease, and other conditions that influence hematological parameters. Explainable AI techniques will be integrated to improve model transparency and interpretability. Furthermore, different feature selection techniques can be explored to assess their impact on accuracy, and additional machine learning frameworks, including deep learning methods, may be tested to refine performance , followed by assessment of the results using multiple evaluation metrics. Further improvements will involve integrating data from other hemoglobinopathies, which will contribute to a more comprehensive classification system, ultimately enabling the model’s deployment in clinical settings to support thalassemia diagnosis and management.

### Research ethics

This study was a retrospective analysis utilizing medical records to collect hematological data. Ethical approval was obtained from the Ethics Committee of Ahvaz Jundishapur University of Medical Sciences (AJUMS), with the approval code IR.AJUMS.REC.1402.311. The study involved the use of unidentified data from existing medical records. Confidentiality and data protection standards were strictly maintained throughout the research process.

## Data Availability

In line with our commitment to open science and transparency, the datasets collected and analyzed during this study are not publicly available but can be obtained from the corresponding author upon reasonable request.
